# Anion-assisted amidinium exchange and metathesis†

**DOI:** 10.1039/d2cc03425e

**Published:** 2022-08-23

**Authors:** Oleg Borodin, Yevhenii Shchukin, Jonas Schmid, Max von Delius

**Affiliations:** Institute of Organic Chemistry, Ulm University, Albert-Einstein-Allee 11 89081 Ulm Germany max.vondelius@uni-ulm.de

## Abstract

Dynamic covalent chemistry has become an invaluable tool for the design and preparation of adaptable yet robust molecular systems. Herein we explore the scope of a largely overlooked dynamic covalent reaction – amidinium exchange – and report on conditions that allow formal amidinium metathesis reactions.

## Abstract 2

In this article, we explore the scope of a largely overlooked dynamic covalent reaction – amidinium exchange – and report on conditions that allow formal amidinium metathesis reactions.

## Graphical abstract

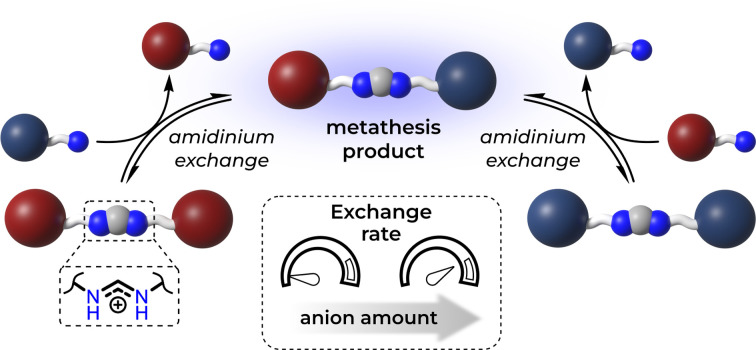

Development of new dynamic covalent chemistries (DCvC) benefits a wide range of research areas such as materials science,^1–4^ bioconjugation,^5,6^ stereoselective synthesis,^7^ (bio)molecular sensing,^8,9^ and the construction of mechanically interlocked architectures (MIAs).^10^ Dynamic covalent reactions (DCRs) involving primary amines are especially important due to the ubiquity of this class of organic compounds. The reversible formation and exchange reactions of imines, aminals, and amides are classical examples of amine-based DCRs.^11^ While these reactions are being constantly studied and advanced,^12,13^ there is a number of new (rediscovered) DCRs, which utilize primary amines; these new chemistries involve imides,^14^ carbamates,^15^ ureas,^16^ guanidines,^17^ and sulfonamides.^18^ A remarkable example of an amine-based DCR was reported in 2011 by Petitjean and coworkers who recognized the dynamic covalent nature of amidines.^19^ In this first report, the authors focused on the exchange of aryl amines with *N*,*N*′-diarylformamidines and demonstrated that this DCR could be used for the carboxylate-templated assembly of macrocycles.^19^ It occurred to us that amidines and their amidinium salts are widely used in coordination chemistry,^20^ biomedical science,^21,22^ advanced materials,^23,24^ and supramolecular chemistry,^25,26^ and therefore deserve a wider exploration from the perspective of DCvC.

Recently, we showed that amidinium exchange can be exploited for the synthesis of MIAs (*i.e.*, [2]rotaxanes) and unique dynamic combinatorial libraries with kinetically trapped constituents.^27^ We realized, however, that the basic principles that govern amidinium exchange remain largely obscure thus precluding the realization of the full potential of this DCR. In particular, we felt that the substrate scope, solvent effects and most importantly, the ability of different anions to affect the rate of exchange could be better understood.

Herein we report on two notable features of amidinium exchange that render this DCR a promising tool in biomedical research and studies of chemical networks. First, we found that besides a wide range of organic solvents amidinium exchange can take place in aqueous media, at least under certain conditions. Second, we discovered the extraordinary ability of anions to modulate the kinetics of amidinium exchange and formal metathesis‡‡Consistent with precedence in the field of DCvC,^28,29^ we use the term “metathesis” in a strictly formal way to indicate a reaction of type “AA + BB ⇄ AB” without making any claim about the reaction mechanism. (Fig. 1B). In recent years, kinetic effects in dynamic covalent systemshave been receiving increased attention due to a shift of interest towards out-of-equilibrium networks.^30–35^ The anion-specific modulation of the kinetics of (amidinium-based) dynamic covalent reactions may therefore become a tool for controlling the behavior of complex networks.

**Fig. 1:**
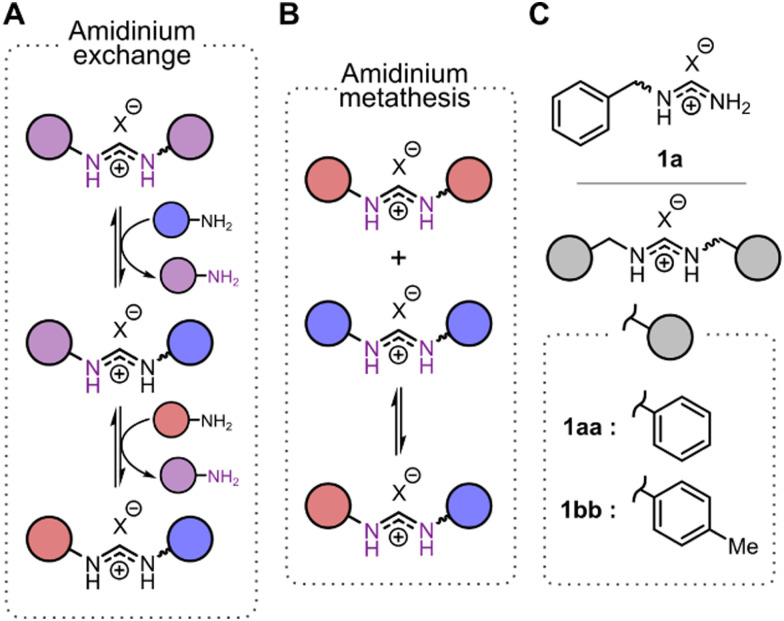
(A) General scheme of the amidinium exchange. (B) General scheme of the amidinium metathesis. (C) Key amidinium species studied in this work.

To obtain further insights into amidinium exchange,^19^ we set out to study a model reaction between the simplest unsubstituted formamidinium ion (FA) and benzylamine (BnNH_2_). The tetraphenylborate salt of FA already played the central role in our previous preparation of MIAs.^27^BnNH_2_ is a cheap, water-soluble moderately basic aliphatic amine that is convenient to monitor by HPLC. The reaction between FA and BnNH_2_ yielded an equilibrium mixture of two compounds – the product of single exchange (1a) and the product of double exchange (1aa) (Fig. 1C). First, we performed this reaction in a range of solvents of different polarity and hydrogen bond donating/accepting ability (Section 3.2, ESI†). Typical equilibration times at room temperature were between 40 and 70 minutes and did not depend on the solvent polarity. The fastest equilibration (40 min) was observed in acetonitrile (MeCN) or ethyl acetate as a solvent. Interestingly, protic solvents (*e.g.*, methanol or aqueous THF with 20% v/v H_2_O) significantly decreased the exchange rate, possibly due to hydrogen bonding between solvent and BnNH_2_, which decreased the nucleophilicity of the amine.

Next, we wanted to compare the performance of different starting materials and essentially replace FA-BPh_4_ with amidinium ions featuring amines of different basicities and reactivities, *i.e.*, benzylic (1bb), aliphatic (1dd), and aromatic (1cc) amines (Fig. 2A). At this stage of the study, we used formamidinium salts with weakly coordinating anions, in order to exclude the influence of anion-binding on the kinetics and thermodynamics of the exchange reactions. In MeCN, the simplest amidinium ion (FA) exhibited the fastest exchange kinetics and afforded the most complete conversion (*ca.* 60%) towards the product of double exchange 1aa (Fig. 2B). Amidinium substrates 1bb and 1dd showed slower exchange rates compared to FA and afforded only 10–20% conversion to 1aa. The outlier kinetics observed for the formation of 1aa from 1cc are likely a consequence of the outlier acidity of 1cc and 1ac.^19^ The increasing equilibrium concentration of 1aa in the row 1dd–1bb–1cc is reciprocally proportional to the basicities of the corresponding amine leaving groups (the less basic amine being the better leaving group). The exceptionally large amount of 1aa formed from FA represents a clear outlier that is due to the ability of NH_3_ to escape the reaction mixture^27^ and therefore is more of a kinetic rather than a thermodynamic effect.

**Fig. 2:**
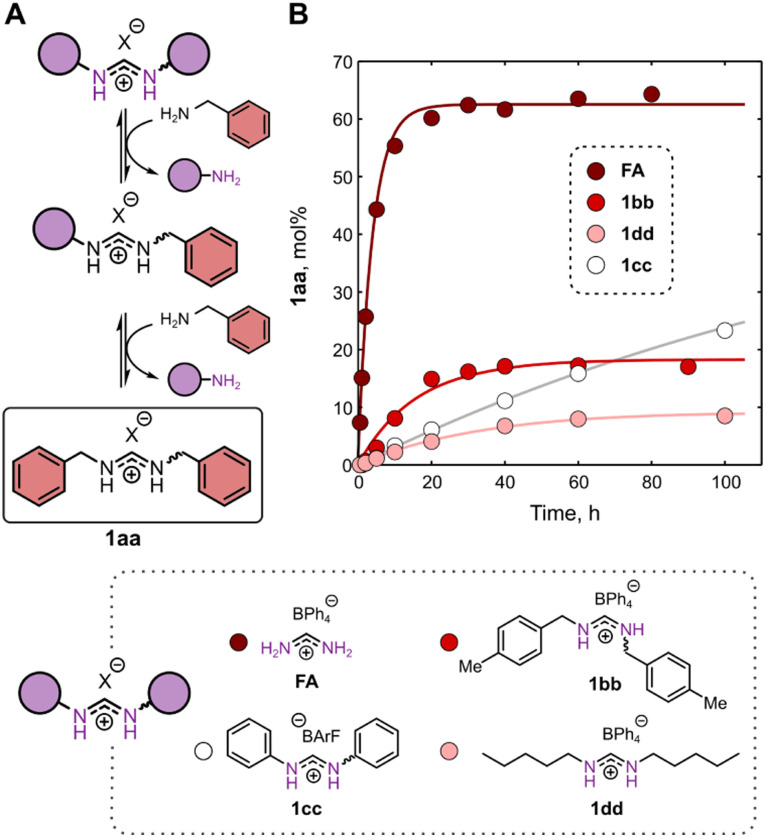
Effect of the amidinium substrate on the exchange rate and the product distribution. (A) Reaction scheme. (B) Monitoring the formation of the two-fold exchange product (1aa) from four different amidinium salts. The lines are shown to guide the eye. Reaction conditions: 50 mM amidinium salt (FA/1bb/1cc/1dd), 100 mM BnNH_2_, solvent: MeCN; room temperature. Details of the experimental procedures can be found in ESI,† Section 3.1.

Having identified the simplest unsubstituted formamidinium ion as the most reactive starting material and knowing that some of its commercially available salts are well-soluble in water, we wondered if amidinium exchange could be carried out in aqueous media, despite the risk of competing and irreversible hydrolysis. Indeed, a reaction between formamidinium acetate (FA-OAc) and BnNH_2_ in pure water at room temperature afforded a mixture of 1a and 1aa, where the former was the major product (Fig. S9, ESI†). Similar outcomes were observed in aqueous buffers in the pH range from 5.5 to 9.5 (Fig. S10, ESI†). With respect to competing hydrolysis to formamides, this dynamic covalent system exhibited unusual behavior: while 1aa reached equilibrium and its amount remained constant (yet low), 1a was metastable and slowly hydrolysed over time. The hydrolysis was especially pronounced at pH 11.5 (Fig. S10, ESI†). The water solubility and transient nature of 1a and similar mono-*N*-substituted formamidinium ions make these molecules interesting candidates for the use in drug delivery and out-of-equilibrium systems.

To test whether the counterion had any influence on amidinium exchange, we performed the exchange reaction with FA-OAc in those solvents that were previously used for the amidinium salt FA-BPh_4_ (wherever the solubility of FA-OAc allowed) (Table S2 and Fig. S11, S12; ESI†). Besides slight variations in the composition of the equilibrium mixture, we observed a moderately increased exchange rate. This finding indicates that acetate as a weakly basic anion affects the kinetics of amidinium exchange. Our observation is corroborated by computational studies by Petitjean and coworkers who showed that a carboxylate ion served as a proton shuttle in the mechanism proposed for the exchange of *N*,*N*′-diarylsubstituted formamidinium ions.^19^

One of the key features of compounds with dynamic covalent bonds is their ability to undergo metathesis reactions.^34,36–39^ This type of DCR has been widely used in numerous fields including the synthesis of polymers as well as advanced molecular architectures and the exploration of complex chemical systems.^1,40–44^ Therefore, we decided to test if *N*,*N*′-disubstituted formamidinium ions were able to undergo formal metathesis.‡ To our delight, the reaction between 1aa and 1bb did take place and afforded the metathesis product 1ab (Fig. 3). The hydrolysis of the amidinium salts at room temperature did not produce sufficient amounts of BnNH_2_ to initiate the reaction§§Hydrolysis of the starting materials – 1aa and 1bb – results in formation of either BnNH_2_ or 4-methylbenzylamine, respectively. However, the amidinium metathesis did not take place until at least 2.5 mol% BnNH_2_ was added to the reaction mixture. This clearly indicates that the hydrolysis of the starting amidinium salts did not generate enough benzylamines to make the metathesis happen at observable rates. (although this scenario is common for some other metathesis reactions, *e.g.*, (trithio)orthoester^37,39^ or imine^29,34^ metathesis) (Fig. 3A). Therefore, we used externally added BnNH_2_ and found that the reaction was substantially accelerated upon increasing the catalytic amount of BnNH_2_ (Fig. 3B, C and Fig. S13, ESI†).

**Fig. 3:**
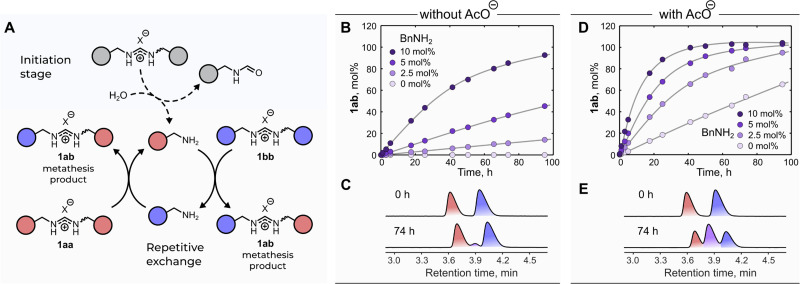
(A) Reaction cycle of the formal amidinium metathesis‡ studied in the present work. In the absence of acetate, hydrolysis of the amidinium salts under standard reaction conditions did not afford sufficient amounts of the amines to initiate the exchange cycle. (B and D) Kinetic plots: formation of metathesis product 1ab in the absence (B) or presence (D) of AcO^−^ (1aa and 1bb were used in the form of BPh_4_-salts). Relative amounts of BnNH_2_ are indicated with respect to the total amount of the amidinium species. Relative amount of 1ab (mol%) was calculated with respect to the initial amount of either 1aa or 1bb (which were used as an equimolar mixture). The lines are shown to guide the eye. (C and E) HPLC traces at 0 h and 74 h of the amidinium metathesis (with 2.5 mol% BnNH_2_). The purple peak denotes metathesis product 1ab. Reaction conditions: 25 mM 1aa, 25 mM 1bb, 50 mM NBu_4_OAc, 0–5 mM BnNH_2_, solvent: MeCN; room temperature. Details of the experimental procedures can be found in ESI,† Section 4.1.

Having in mind that anions affect the amidinium exchange rate, we were curious about anion effects in the metathesis reaction. By adding one equivalent of NBu_4_OAc to a mixture of 1aa and 1bb, we observed a dramatic increase in the metathesis rate (Fig. 3D, E and Fig. S14, Table S3, ESI†). To our surprise, even in the absence of BnNH_2_ the reaction took place at a reasonable rate. We attributed the latter observation to the ability of the carboxylate to accelerate the hydrolysis of 1aa/1bb thus leading to generation of BnNH_2_ (for supporting experimental evidence, see Fig. S15, ESI†). Intrigued by this finding, we decided to test if carboxylate ions can be used to ramp up the metathesis rate on demand. We launched the reaction between 1aa and 1bb in the presence of 4 mol% BnNH_2_ and observed its very slow progression over one day. Next, we added different substoichiometric amounts of acetate and continued the reaction monitoring. As expected, the reaction significantly accelerated and, more importantly, its equilibration rate depended on the acetate amount (Fig. 4A and Fig. S16, ESI†). This finding showcases the potential of anionic stimuli for fine-tuning the kinetic parameters of complex dynamic chemical systems approaching equilibrium.

**Fig. 4:**
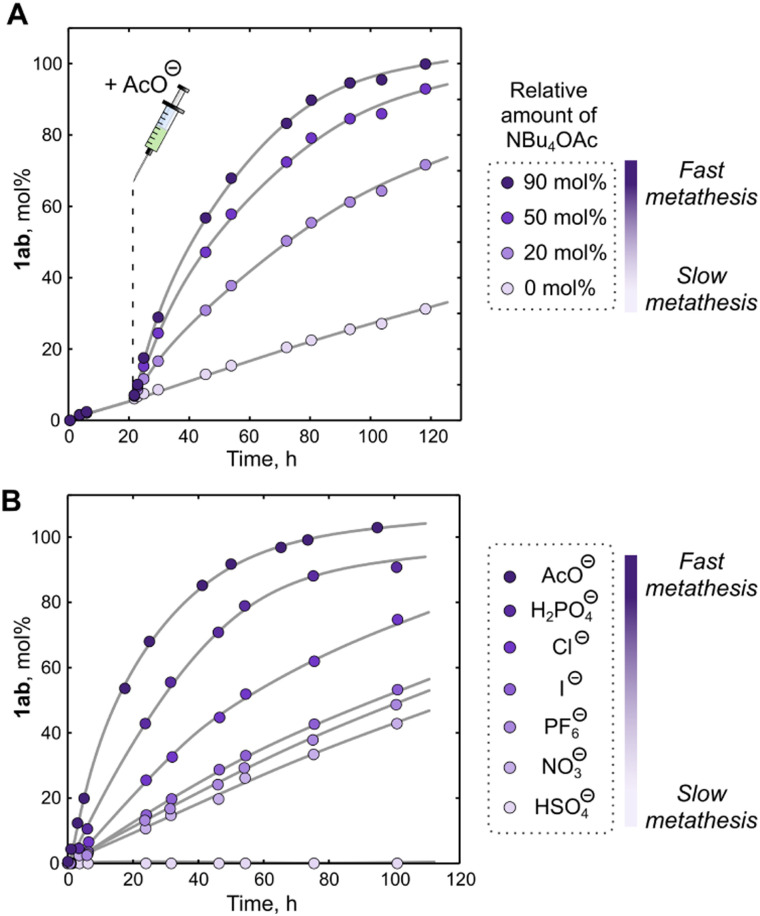
Kinetic plots of the formal amidinium metathesis‡ in an equimolar mixture of 1aa and 1ab controlled by anions (solvent – MeCN, room temperature, total concentration of the amidinium species – 50 mM). All experiments were carried out in the presence of catalytic amounts of BnNH_2_ (4–5 mol%). Relative amount of metathesis product 1ab (mol%) was calculated with respect to the initial amount of either 1aa or 1bb (which were used as an equimolar mixture). (A) Kinetic plots showing dramatic increase of the metathesis rate upon addition of different amounts of the acetate. (B) Kinetic plots comparing formation of the metathesis product in the presence of 5 mol% BnNH_2_ and different anions (as NBu_4_^+^ salts, 100 mol%). All relative amounts (besides the amount of 1ab) are with respect to the total amount of the amidinium species. The lines are shown to guide the eye. Details of the experimental procedures and full kinetic plots can be found in ESI,† Sections 4.1 and 4.3.

Finally, we tested other simple anions as potential modulators of the metathesis kinetics. The chloride ion and especially the dihydrogen phosphate ion did increase the reaction rate, even in a solvent (MeCN) containing 10–20% (v/v) MeOH and despite poor solubility of the amidinium-phosphate complexes (Fig. 4B and Fig. S17, Table S3, ESI†). Other anions such as I^−^, NO_3_^−^, and PF_6_^−^ did not significantly alter the metathesis kinetics, possibly due to their lower basicity preventing them to act as efficient proton shuttle during amidinium exchange. Interestingly, hydrogen sulfate fully suppressed the metathesis reaction, which we attribute to the relatively strong acidity of HSO_4_^−^ (p*K*_a_ = 1.99).^45^ This anion can thus fully protonate benzylic amines – key metathesis intermediates – and thereby renders them unreactive. Although dihydrogen phosphate (p*K*_a_ = 7.20)^45^ also protonates the amines, the equilibrium concentration of the unprotonated amine was still sufficient to drive the amidinium metathesis.

In conclusion, we identified the simplest unsubstituted formamidinium ion as the most reactive substrate for amidinium exchange and explored the solvent scope of the reaction. Amidinium exchange can be carried out with substrate FA-OAc in aqueous medium (either in pure water or a buffer in the pH range of 5.5–9.5), indicating potential uses of this underexplored DCR in biomedical studies. Furthermore, we showed a rare case of control over the kinetics of a dynamic covalent reaction by addition of certain anions (as NBu_4_^+^ salts). Our results establish amidinium exchange as a platform for anion sensing and kinetically gated chemical networks.

This work was supported by the Deutscher Akademischer Austauschdienst (DAAD, PhD scholarship to O. B.), the Deutsche Forschungsgemeinschaft (DFG, Emmy-Noether Grant DE1830/2-1), and the European Research Council (ERC Starting Grant 802428 – SUPRANET).

## Conflicts of interest

There are no conflicts to declare.

## Supplementary Material

CC-058-D2CC03425E-s001

## References

[cit1] Tretbar C. A., Neal J. A., Guan Z. (2019). J. Am. Chem. Soc..

[cit2] Van Lijsebetten F., Holloway J. O., Winne J. M., Du Prez F. E. (2020). Chem. Soc. Rev..

[cit3] König N. F., Mutruc D., Hecht S. (2021). J. Am. Chem. Soc..

[cit4] Rivero D. S., Paiva-Feener R. E., Santos T., Martín-Encinas E., Carrillo R. (2021). Macromolecules.

[cit5] Ruff Y., Garavini V., Giuseppone N. (2014). J. Am. Chem. Soc..

[cit6] Diehl K. L., Kolesnichenko I. V., Robotham S. A., Bachman J. L., Zhong Y., Brodbelt J. S., Anslyn E. V. (2016). Nat. Chem..

[cit7] Walsh M. P., Phelps J. M., Lennon M. E., Yufit D. S., Kitching M. O. (2021). Nature.

[cit8] Herrmann A. (2014). Chem. Soc. Rev..

[cit9] Lafuente M., Solà J., Alfonso I. (2018). Angew. Chem., Int. Ed..

[cit10] Kandrnálová M., Kokan Z., Havel V., Nečas M., Šindelář V. (2019). Angew. Chem., Int. Ed..

[cit11] Jin Y., Yu C., Denman R. J., Zhang W. (2013). Chem. Soc. Rev..

[cit12] Erguven H., Keyzer E. N., Arndtsen B. A. (2020). Chem. – Eur. J..

[cit13] Carbajo D., Ruiz-Sánchez A. J., Nájera F., Pérez-Inestrosa E., Alfonso I. (2021). Chem. Commun..

[cit14] Fang Q., Zhuang Z., Gu S., Kaspar R. B., Zheng J., Wang J., Qiu S., Yan Y. (2014). Nat. Commun..

[cit15] Septavaux J., Tosi C., Jame P., Nervi C., Gobetto R., Leclaire J. (2020). Nat. Chem..

[cit16] Ying H., Zhang Y., Cheng J. (2014). Nat. Commun..

[cit17] Melchor Bañales A. J., Larsen M. B. (2020). ACS Macro Lett..

[cit18] Hai Y., Zou H., Ye H., You L. (2018). J. Org. Chem..

[cit19] Capela M. d, Mosey N. J., Xing L., Wang R., Petitjean A. (2011). Chem. – Eur. J..

[cit20] Cotton F., Daniels L. M., Maloney D. J., Matonic J. H., Murillo C. A. (1994). Polyhedron.

[cit21] Kumamoto T. , in Amidines and Guanidines in Natural Products and Medicines, John Wiley Sons, Ltd, 2009, ch. 10, pp. 295–313

[cit22] Sun J., He L., Gao Y., Zhai L., Ji J., Liu Y., Ji J., Ma X., Mu Y., Tang D., Yang H., Iqbal Z., Yang Z. (2021). Mendeleev Commun..

[cit23] Wulff G., Schönfeld R. (1998). Adv. Mater..

[cit24] Ren X., Wang X., Sun Y., Chi X., Mangel D., Wang H., Sessler J. L. (2019). Org. Chem. Front..

[cit25] Yamada H., Wu Z.-Q., Furusho Y., Yashima E. (2012). J. Am. Chem. Soc..

[cit26] Kohlhaas M., Zähres M., Mayer C., Engeser M., Merten C., Niemeyer J. (2019). Chem. Commun..

[cit27] Borodin O., Shchukin Y., Robertson C. C., Richter S., von Delius M. (2021). J. Am. Chem. Soc..

[cit28] Caraballo R., Rahm M., Vongvilai P., Brinck T., Ramström O. (2008). Chem. Commun..

[cit29] Ciaccia M., Pilati S., Cacciapaglia R., Mandolini L., Di Stefano S. (2014). Org. Biomol. Chem..

[cit30] Lehn J.-M. (2015). Angew. Chem., Int. Ed..

[cit31] Miljanić O. (2017). Chem.

[cit32] Heinen L., Walther A. (2019). Sci. Adv..

[cit33] Larsen D., Beeren S. R. (2019). Chem. Sci..

[cit34] Schaufelberger F., Seigel K., Ramström O. (2020). Chem. – Eur. J..

[cit35] Kriebisch C. M. E., Bergmann A. M., Boekhoven J. (2021). J. Am. Chem. Soc..

[cit36] Stephenson N. A., Zhu J., Gellman S. H., Stahl S. S. (2009). J. Am. Chem. Soc..

[cit37] Brachvogel R.-C., von Delius M. (2015). Chem. Sci..

[cit38] Fritze U. F., von Delius M. (2016). Chem. Commun..

[cit39] Bothe M., Orrillo A. G., Furlan R. L., von Delius M. (2019). Synlett.

[cit40] Behera P. K., Raut S. K., Mondal P., Sarkar S., Singha N. K. (2021). ACS Appl. Polym. Mater..

[cit41] Jiang X., Laffoon J. D., Chen D., Pérez-Estrada S., Danis A. S., Rodríguez-López J., Garcia-Garibay M. A., Zhu J., Moore J. S. (2020). J. Am. Chem. Soc..

[cit42] Colomer I., Borissov A., Fletcher S. P. (2020). Nat. Commun..

[cit43] Balakrishna B., Menon A., Cao K., Gsänger S., Beil S. B., Villalva J., Shyshov O., Martin O., Hirsch A., Meyer B., Kaiser U., Guldi D. M., von Delius M. (2020). Angew. Chem., Int. Ed..

[cit44] Howlett M. G., Scanes R. J. H., Fletcher S. P. (2021). JACS Au.

[cit45] Harris D. C. , Quantitative Chemical Analysis, New York, NY, W. H. Freeman and Company, 8th edn, 2010

