# Supplementary material for: Anion-assisted amidinium exchange and metathesis
Source: Chem Commun (Camb). 2022 Aug 23;58(73):10178–81. doi: 10.1039/d2cc03425e (PMC9469691; doi:10.1039/d2cc03425e)
Supplement: CC-058-D2CC03425E-s001 [file CC-058-D2CC03425E-s001.pdf]

Electronic Supplementary Material (ESI) for ChemComm.
This journal is © The Royal Society of Chemistry 2022

Electronic supplementary information

Anion-assisted amidinium exchange and metathesis

Oleg Borodin,[a] Yevhenii Shchukin,[a] Jonas Schmid,[a] and Max von Delius[a]

a) Institute of Organic Chemistry, Ulm University, Albert-Einstein-Allee 11, 89081 Ulm, Germany.

E-Mail: max.vondelius@uni-ulm.de

Contents

1 General methods and abbreviations **2**

2 Synthesis of amidinium salts **3**

3 Amidinium exchange: substrate and solvent scope **4**

3.1 General procedure 4

3.2 Amidinium exchange with FA-BPh_4: solvent scope 5

3.3 Substrate scope 8

3.4 Amidinium exchange with FA-OAc: solvent scope 8

4 Amidinium metathesis **11**

4.1 General procedure 11

4.2 Effect of the primary amine amount on the metathesis rate 12

4.3 Effect of anions on the metathesis rate 13

5 NMR spectra **17**

References **18**

1 General methods and abbreviations

All commercially available reagents and solvents were purchased from Sigma Aldrich, Alfa Aesar, ACROS Organics, TCI, Fluorochem or VWR and were used without further purification. NMR spectra were recorded on Bruker Avance 400 (^{1}H: 400 MHz; ^{13}C: 101 MHz) spectrometers at room temperature and referenced to the residual solvent peak (^{1}H: $CDCl_3$, 7.26 ppm; CD_3CN, 1.94 ppm, DMSO-d_6, 2.50 ppm; CD_2Cl_2, 5.32 ppm; ^{13}C: $CDCl_3$, 77.16 ppm; CD_3CN, 1.32 ppm, DMSO-d_6, 39.52 ppm, CD_2Cl_2, 53.84 ppm). Chemical shifts (δ) are denoted in ppm. High-resolution mass spectra were recorded on an Agilent QTOF 6546 mass spectrometer (ESI, positive polarity; solvent: acetonitrile).

LCMS reaction monitoring

All samples for LCMS analysis were prepared by diluting 1-3 μL of the analyzed solution (e.g., a reaction mixture) in 1 mL of LCMS-grade acetonitrile. This extent of dilution was sufficient to stop (or significantly slow down) dynamic covalent exchange in order to reliably quantify the analytes by HPLC. LCMS analysis of amidinium exchange and metathesis was performed on Shimadzu LCMS-2020 using Ascentis® C8 HPLC column (10 cm × 4.6 mm, particle size – 3 μm) or Kinetex® C18 HPLC column (10 cm × 4.6 mm, particle size – 2.6 m). Ascentis® C8 HPLC column was used for all samples containing *N*,*N'*-di(4-methylbenzyl)formamidinium (**1bb**).

HPLC method. Isocratic elution was applied using: a) 94% mobile phase B and 6% mobile phase A at 50 °C and flow rate 1.0 mL/min (for Kinetex® C18 HPLC column) and b) 96% mobile phase B and 4% mobile phase A at 50 °C and flow rate 1.0 mL/min (for Ascentis® C8 HPLC column). Mobile phase A: 0.023 M HCO_2NH_4 and 0.0019 M HCO_2H in H_2O. Mobile phase B: acetonitrile. HPLC monitoring was performed with a photodiode array detector at λ= 220 nm.

List of abbreviations

BArF	tetrakis[3,5-bis(trifluoromethyl)-phenyl]borate
$BnNH_2$	benzylamine
BPh_4^-	tetraphenylborate
DMSO	dimethyl sulfoxide
eq.	equivalents
EtOAc	ethyl acetate
HPLC	high-performance liquid chromatography
LCMS	liquid chromatography – mass spectrometry
MeCN	acetonitrile
OAc	acetate
PhMe	toluene
r.t.	room temperature
THF	tetrahydrofuran

2 Synthesis of amidinium salts

Amidinium salts **1aa**·BPh_4, **1cc**·BArF, **1dd**·BPh_4, and **FA**·BPh_4 were synthesized according to previously published procedures.[1]

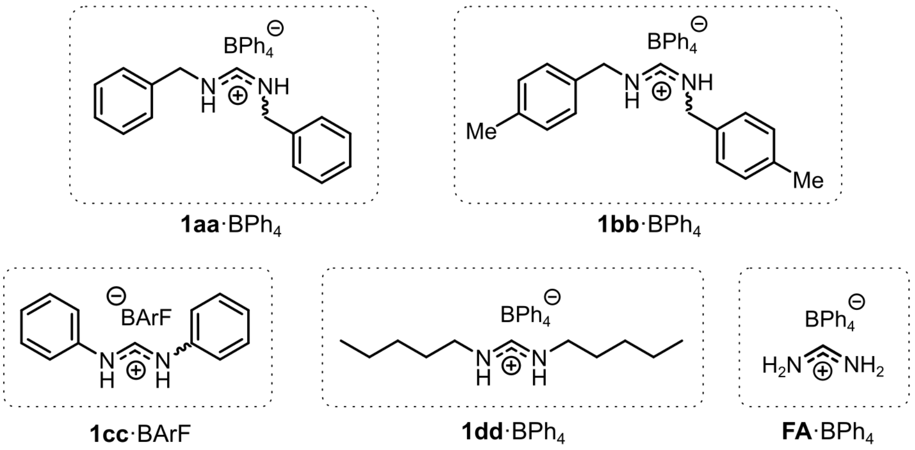

Figure S1: Amidinium salts used in the present work

***N,N′*-di(4-methylbenzyl)formamidinium tetraphenylborate (1bb·BPh$_4$)**

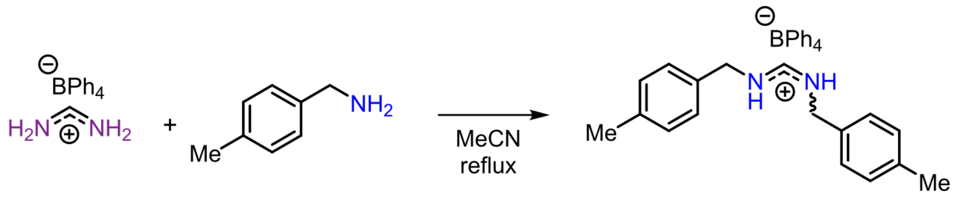

Formamidinium tetraphenylborate (**FA**·BPh_4) (0.3 mmol, 1.0 eq.) was dissolved in MeCN (4 mL), and 4-methylbenzylamine (0.8 mmol, 2.8 eq.) was added to the solution. The reaction mixture was stirred under reflux for 30 min and all volatiles were then removed under reduced pressure. The residue was re-dissolved in MeCN (0.75 mL) and PhMe (7.5 mL) was added to the mixture. The solution was kept at +4 °C overnight and the resulting crystals were filtered off to afford the desired products **1bb**·BPh_4 as colorless crystals (0.10 g, 0.17 mmol, 65%). For preparation of the analytically pure sample (without traces of 4-methylbenzylamine), the product was recrystallized twice by dissolving it in the minimal volume of MeCN/CH_2Cl_2 mixture (1:1) and adding 5-10 fold volume of PhMe.

^{1}H NMR (400 MHz, CD_3CN, 295 K)* δ 7.69 (s, 1H, CH amidinium), 7.33 – 7.11 (m, 16H, CH Ar: BPh_4 + amidinium Ph), 7.00 (t, *J* = 7.44 Hz, 8H, CH Ar BPh_4), 6.85 (t, *J* = 7.20 Hz, 4H, CH Ar BPh_4), 4.48 (s, 2H, CH_2 benzylic *E,Z*), 4.43 (bs, 4H, CH_2 benzylic *E,E*), 4.35 (s, 2H, CH_2 benzylic *E,Z*), 2.34 (s, 6H, CH_3).
*The NMR spectrum contains both isomers of the product – *E,Z* and *E,E*. The integrals of each isomer specified in parentheses are treated independently.

^{13}C NMR (101 MHz, CD_3CN, 298 K) δ 164.80 (q, $^1J_{B\text{-}C}$ = 49.28 Hz, C Ar BPh_4), 155.71, 139.44, 139.36, 136.72 (q, *J* = 1.40 Hz, C Ar BPh_4), 133.43, 131.57, 130.52, 130.43, 128.86, 128.61, 126.62 (q, *J* = 2.69 Hz, C Ar BPh_4), 122.79 (C Ar BPh_4), 51.45, 46.46, 21.15.

HRMS: m/z 253.16993 $[M+H]^+$ (calculated for $C_{17}H_{21}N_2^+$ 253.17080).

3 Amidinium exchange: substrate and solvent scope

3.1 General procedure

A 1.5 mL HPLC vial was charged with a formamidinium salt (40 μmol)* and 1,2,4,5-tetramethylbenzene (as an internal HPLC standard). The mixture was dissolved in LCMS-grade MeCN (800 μL) and benzylamine (2.0 eq., 80 μmol) was rapidly added to the stirred solution via a Hamilton™ syringe. The reaction progress was monitored by LCMS. Amounts of $BnNH_2$, **1a** and **1aa** (in mol% with respect to the initial amount of the starting formamidinium salts) were determined using the internal standard (calibration curves from Figure S2 were used).

*In case of formamidinium acetate, 48 μmol was used and all other reagent and solvent quantities were scaled proportionally.

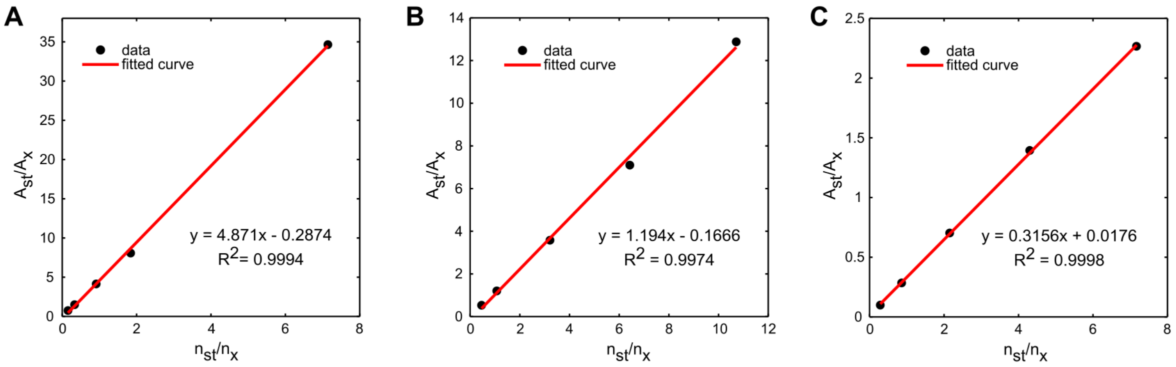

Figure S2: HPLC calibration curves for (A) benzylamine, (B) *N*-benzylformamidinium, (C) *N*,*N*'-dibenzylformamidinium (**1aa**). Internal standard – 1,2,4,5-tetramethylbenzene. n_{st}/n_x – molar ratio between the standard and the calibrated compound; A_{st}/A_x – ratio of chromatographic peak areas of the standard and the calibrated compound. For benzylamine, calibration curves varied depending on the HPLC method (possibly, due to different content of an acidic buffer used, which affected the degree of protonation of **$BnNH_2$** and thus its molar absorptivity); therefore, the curve was updated whenever a new HPLC method was applied.

3.2 Amidinium exchange with FA-BPh$_4$: solvent scope

Table S1: Solvent scope of the amidinium exchange between **FA-BPh$_4$** and **BnNH$_2$**. Reaction conditions: 50 mM **FA-BPh$_4$**, 100 mM **BnNH$_2$**, room temperature.

Entry	Solvent	Equilibration time[a], min	Equilibrium composition[b] **amine**:**1a**:**1aa**, mol%
1	DMSO	70	49:19:57
2	MeCN	40	40:21:62
3	MeOH	>250	60:36:47[c]
4	EtOH	>250	54:28:49[c]
5	*i*PrOH	>250	65:38:47[c]
6	pyridine	50	55:28:47
7	THF	70	50:27:56
8	EtOAc	40	40:14:65
9	THF/H_2O (20% v/v H_2O)	150	74:33:33

[a] The time when all kinetic curves reach the plateau region (meaning that the concentrations of the monitored species do not significantly change anymore).
[b] Composition of the mixture once the equilibrium is reached (at any time > equilibration time).
[c] Composition of the mixture at t = 250 min (not fully equilibrated mixture).

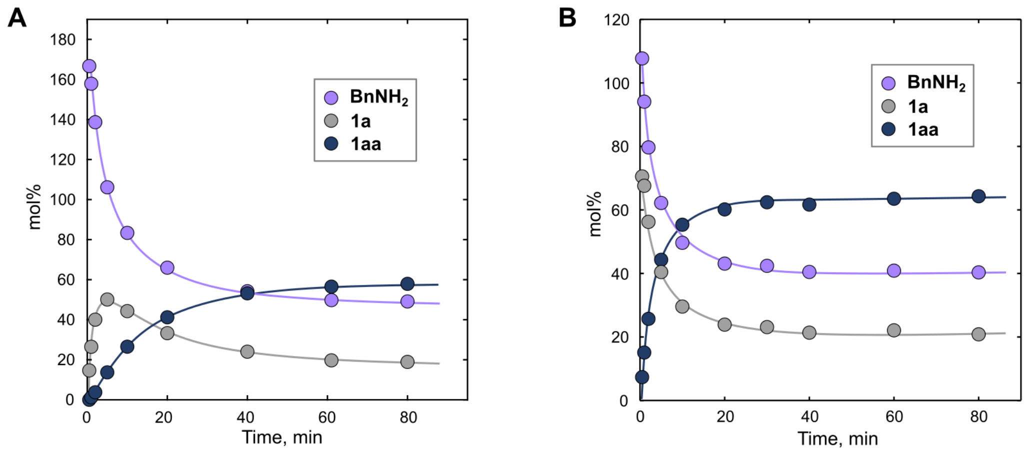

Figure S3: Kinetic plots of the amidinium exchange between **FA-BPh$_4$** and benzylamine in (A) DMSO and (B) MeCN. Reaction conditions: 1.0 eq. **FA-BPh$_4$** (40 µmol), 2.0 eq. **BnNH$_2$** (80 µmol), solvent – 800 µL, r.t. Relative amounts of **BnNH$_2$**, **1a**, and **1aa** (mol%) were calculated with respect to the initial amount of **FA-BPh$_4$**. The lines are shown to guide the eye.

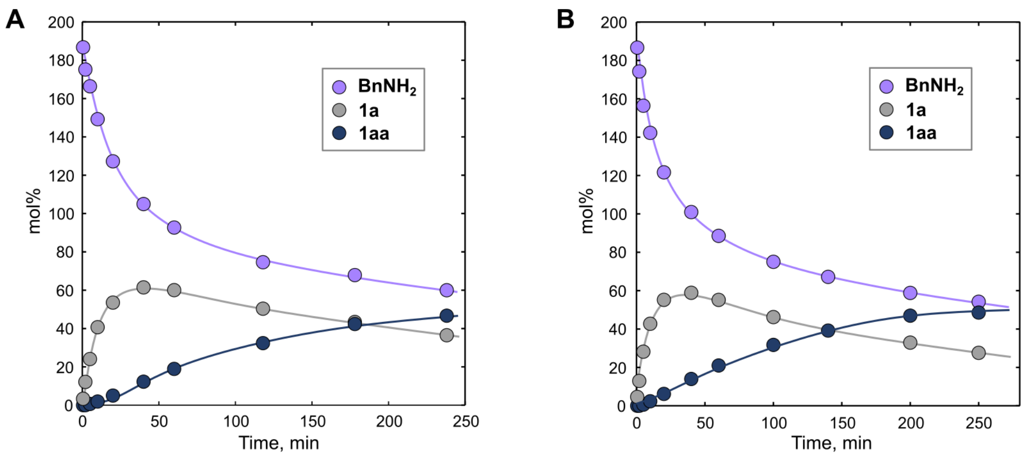

Figure S4: Kinetic plots of the amidinium exchange between **FA-BPh$_4$** and benzylamine in (A) MeOH and (B) EtOH. Reaction conditions: 1.0 eq. **FA-BPh$_4$** (40 µmol), 2.0 eq. **BnNH$_2$** (80 µmol), solvent – 800 µL, r.t. Relative amounts of **BnNH$_2$**, **1a**, and **1aa** (mol%) were calculated with respect to the initial amount of **FA-BPh$_4$**. The lines are shown to guide the eye.

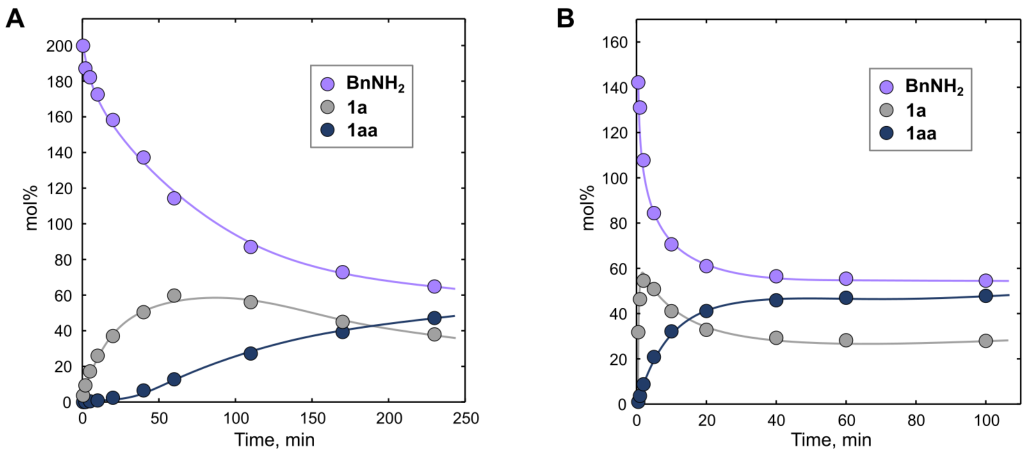

Figure S5: Kinetic plots of the amidinium exchange between **FA-BPh$_4$** and benzylamine in (A) *i*PrOH and (B) pyridine. Reaction conditions: 1.0 eq. **FA-BPh$_4$** (40 µmol), 2.0 eq. **BnNH$_2$** (80 µmol), solvent – 800 µL, r.t. Relative amounts of **BnNH$_2$**, **1a**, and **1aa** (mol%) were calculated with respect to the initial amount of **FA-BPh$_4$**. The lines are shown to guide the eye.

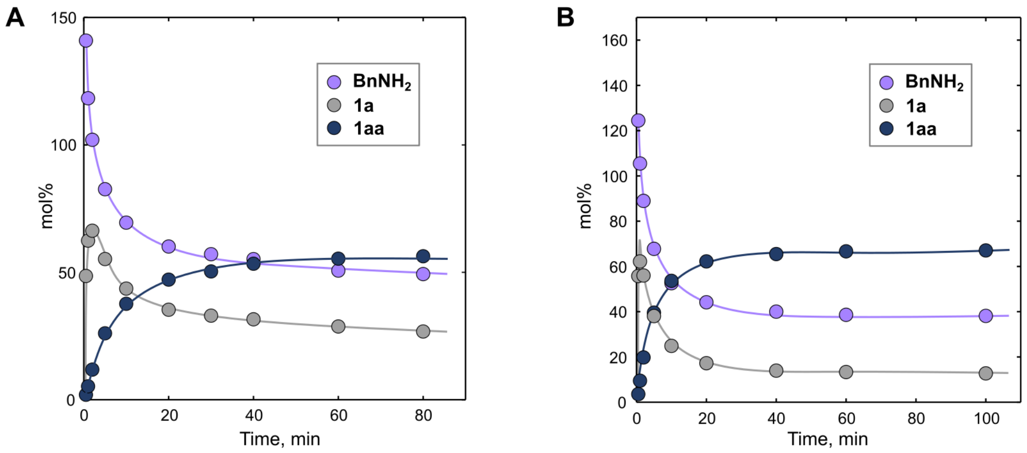

Figure S6: Kinetic plots of the amidinium exchange between **FA-BPh$_4$** and benzylamine in (A) THF and (B) EtOAc. Reaction conditions: 1.0 eq. **FA-BPh$_4$** (40 μmol), 2.0 eq. **BnNH$_2$** (80 μmol), solvent – 800 μL, r.t. Relative amounts of **BnNH$_2$**, **1a**, and **1aa** (mol%) were calculated with respect to the initial amount of **FA-BPh$_4$**. The lines are shown to guide the eye.

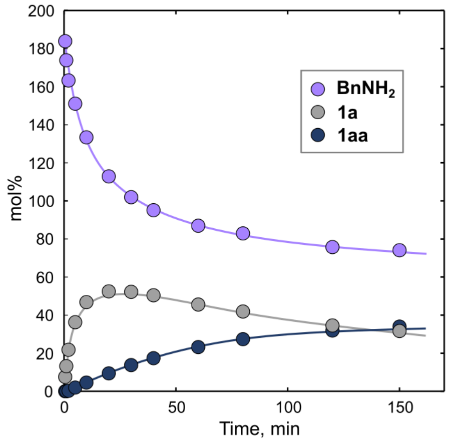

Figure S7: Kinetic plots of the amidinium exchange between **FA-BPh$_4$** and benzylamine in THF/H_2O (20% v/v H_2O). Reaction conditions: 1.0 eq. **FA-BPh$_4$** (40 μmol), 2.0 eq. **BnNH$_2$** (80 μmol), solvent (total volume) – 800 μL, r.t. Relative amounts of **BnNH$_2$**, **1a**, and **1aa** (mol%) were calculated with respect to the initial amount of **FA-BPh$_4$**. The lines are shown to guide the eye.

3.3 Substrate scope

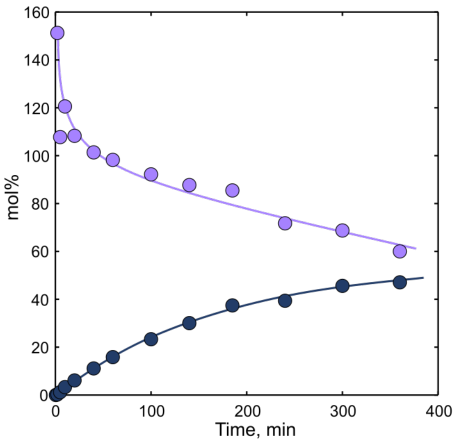

Figure S8: Kinetic plot of the amidinium exchange between benzylamine and **1cc**·BPh_4. Reaction conditions: 1.0 eq. **1cc**·BPh_4 (40 µmol), 2.0 eq. **BnNH₂** (80 µmol), solvent – MeCN (800 µL), r.t. The reaction was monitored by LCMS. Legend: violet – **BnNH₂**, dark blue – **1aa**. The lines are shown to guide the eye.

3.4 Amidinium exchange with FA-OAc: solvent scope

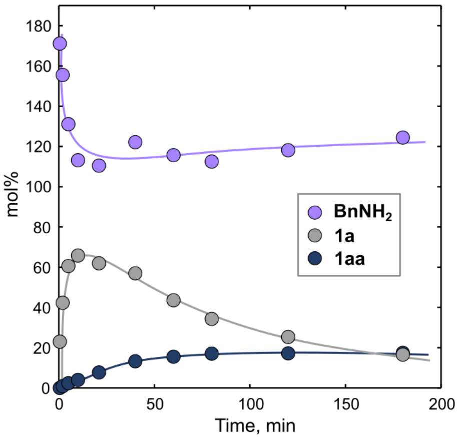

Figure S9: Kinetic plots of the amidinium exchange between **FA-OAc** and benzylamine in pure H_2O. Reaction conditions: 1.0 eq. **FA-OAc** (48 µmol), 2.0 eq. **BnNH₂** (96 µmol), solvent – 960 µL, r.t. The reaction was monitored by LCMS. The lines are shown to guide the eye.

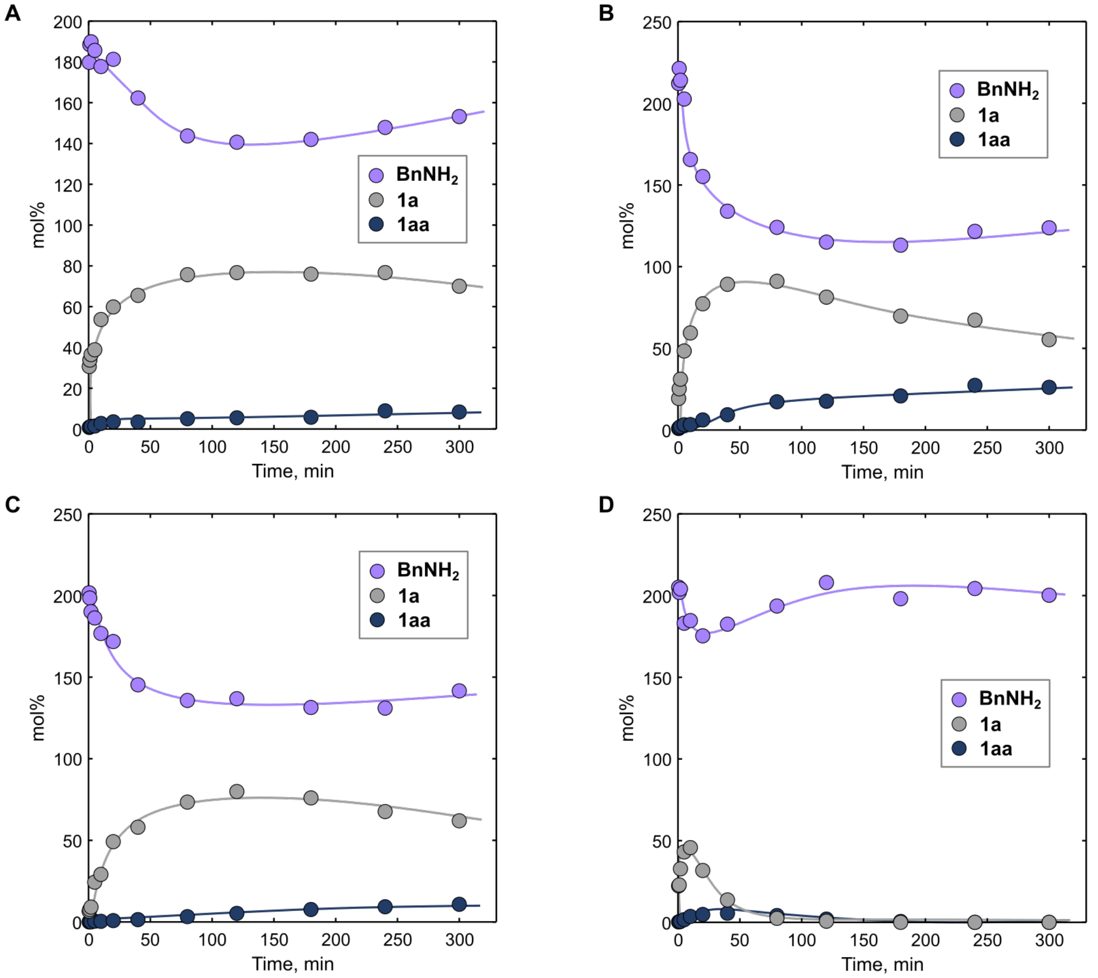

Figure S10: Kinetic plots of the amidinium exchange between **FA-OAc** and benzylamine in H_2O at different pH: A) 5.5 (acetate buffer, 0.2 M); B) 7.5 (phosphate buffer, 0.2 M); C) 9.5 (borate buffer, 0.2 M); D) 11.5 (phosphate buffer, 0.2 M). Reaction conditions: 1.0 eq. **FA-OAc** (50 µmol), 2.0 eq. $\mathbf{BnNH_2}$ (100 µmol), solvent – 2000 µL, r.t. The lines are shown to guide the eye.

Table S2: Solvent scope of the amidinium exchange between **FA-OAc** and $\mathbf{BnNH_2}$ in organic solvents. Reaction conditions: 50 mM **FA-OAc**, 100 mM $\mathbf{BnNH_2}$, room temperature.

Entry	Solvent	Equilibration time[a], min	Equilibrium composition[b] **amine**:**1a**:**1aa**, mol%
1	DMSO	80	42:28:55
2	MeOH	>220	43:38:51[c]
3	*i*PrOH	200	46:34:54
4	THF/H_2O (20% v/v H_2O)	140	62:31:43

[a] The time when all kinetic curves reach the plateau region (meaning that the concentrations of the monitored species do not significantly change anymore).
[b] Composition of the mixture once the equilibrium is reached (at any time > equilibration time).
[c] Composition of the mixture at t = 223 min (not fully equilibrated mixture).

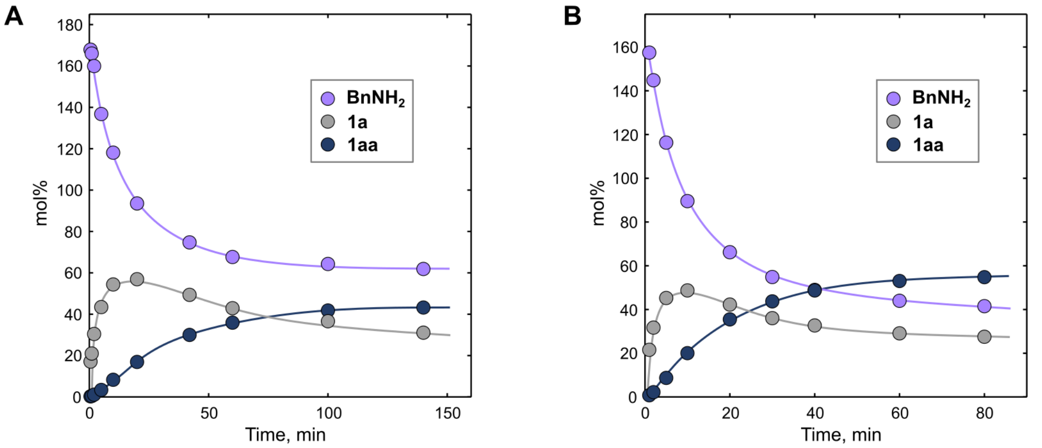

Figure S11: Kinetic plots of the amidinium exchange between **FA-OAc** and benzylamine in (A) THF/H_2O (20% v/v H_2O) and (B) DMSO. Reaction conditions: 1.0 eq. **FA-OAc** (48 μmol), 2.0 eq. **BnNH₂** (96 μmol), solvent – 960 μL, r.t. The reactions were monitored by LCMS. The lines are shown to guide the eye.

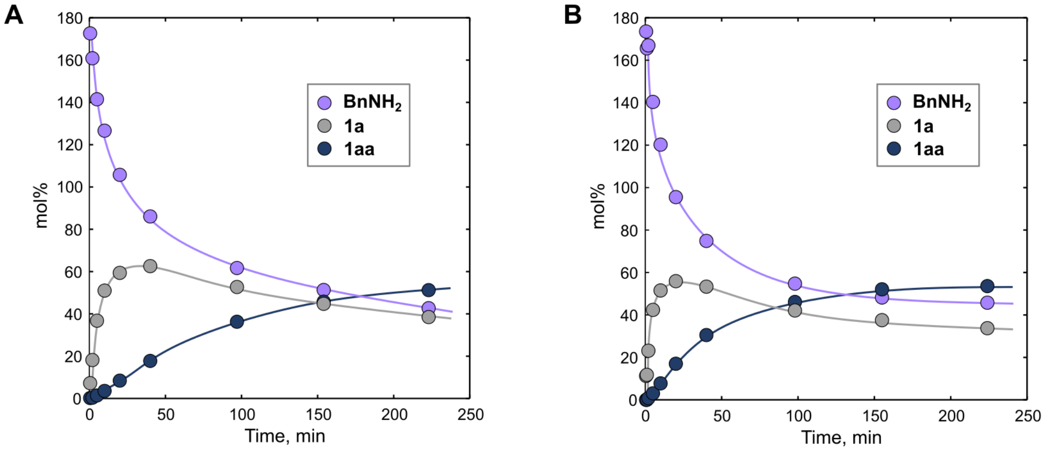

Figure S12: Kinetic plots of the amidinium exchange between **FA-OAc** and benzylamine in (A) MeOH and (B) *i*-PrOH. Reaction conditions: 1.0 eq. **FA-OAc** (48 μmol), 2.0 eq. **BnNH₂** (96 μmol), solvent – 960 μL, r.t. The reactions were monitored by LCMS. The lines are shown to guide the eye.

4 Amidinium metathesis

4.1 General procedure

A 1.5 mL HPLC vial was charged with 1,2,4,5-tetramethylbenzene (as an internal HPLC standard) and equimolar amounts (10 μmol each) of amidinium BPh_4^- salts **1aa** and **1bb** (as stock solutions in MeCN). A stock solution of **$BnNH_2$** in MeCN (56 mM; 0 – 0.10 eq. with respect to the total amount of the amidinium species) was then added. When it was necessary, a stock solution of a tetrabutylammonium salt in MeCN (515 mM; 0 – 1.0 eq. with respect to the total amount of the amidinium species) was subsequently added. The total volume of the reaction mixture (400 μL) was adjusted with MeCN (in fact, this amount of solvent was added to the HPLC vial prior to addition of the reactants). The reaction progress was monitored by LCMS. The amount of **1aa** was determined using the internal standard (the calibration curve from Fig. S2,C was applied). The amount of **1bb** was determined from the HPLC peak area multiplied by a factor which equalized the area of the peaks of **1aa** and **1bb** at t = 0 h (the first HPLC measurement where product **1ab** was not yet formed; equality of the amounts of **1aa** and **1bb** was assured by quantitative NMR of the used stock solutions). Amount of **1ab** was determined from the HPLC peak area assuming that molar absorptivity of **1ab** was a mean of the molar absorptivities of **1aa** and **1bb**. Relative amounts of **1aa**, **1bb**, and **1ab** (mol%) were calculated with respect to the initial amount of either **1aa** or **1bb** (which were used as an equimolar mixture).

4.2 Effect of the primary amine amount on the metathesis rate

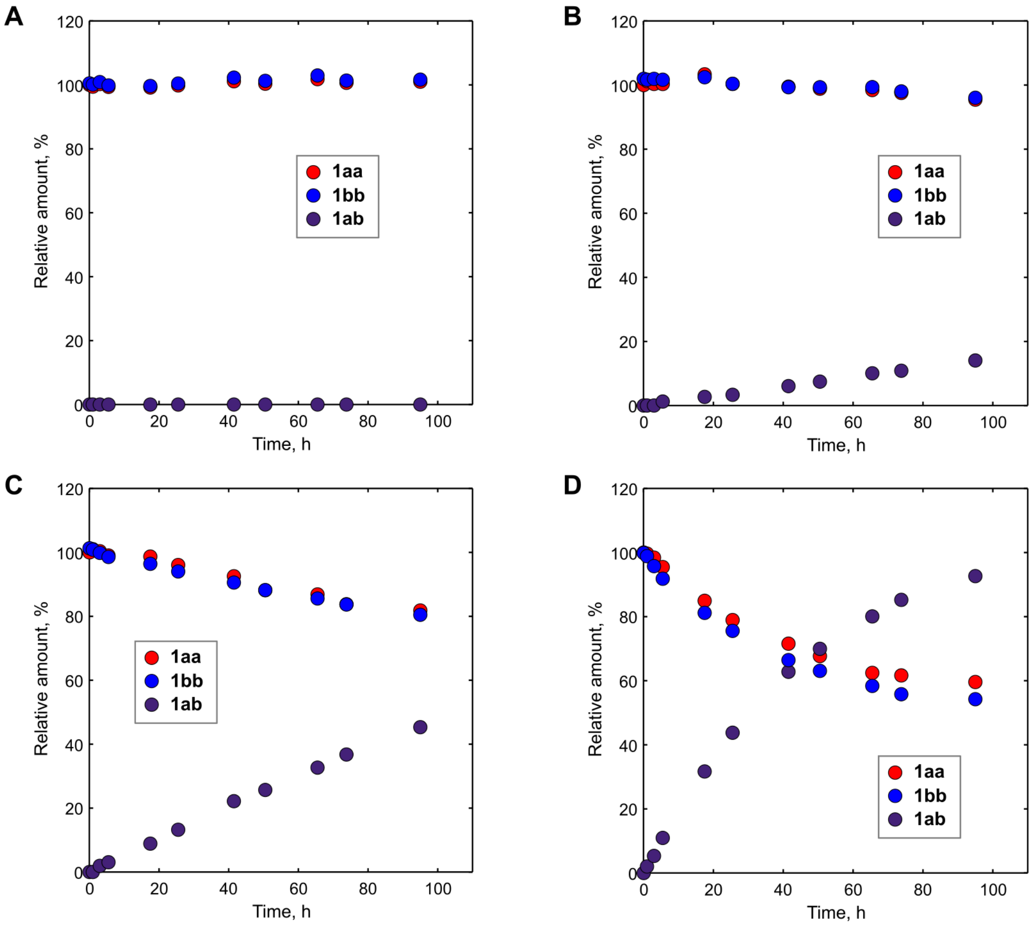

Figure S13: Kinetic plots of the amidinium metathesis between **1aa** and **1bb** in the presence of different amounts of **BnNH$_2$**: A) 0 mol%; B) 2.5 mol%; C) 5 mol%; D) 10 mol% (with respect to the total amount of the amidinium species. Reaction conditions: 1.0 eq. **1aa** (10 μmol), 1.0 eq. **1bb** (10 μmol), 0 – 0.2 eq. **BnNH$_2$** (0–2 μmol), solvent – MeCN (total volume – 400 μL), r.t.

4.3 Effect of anions on the metathesis rate

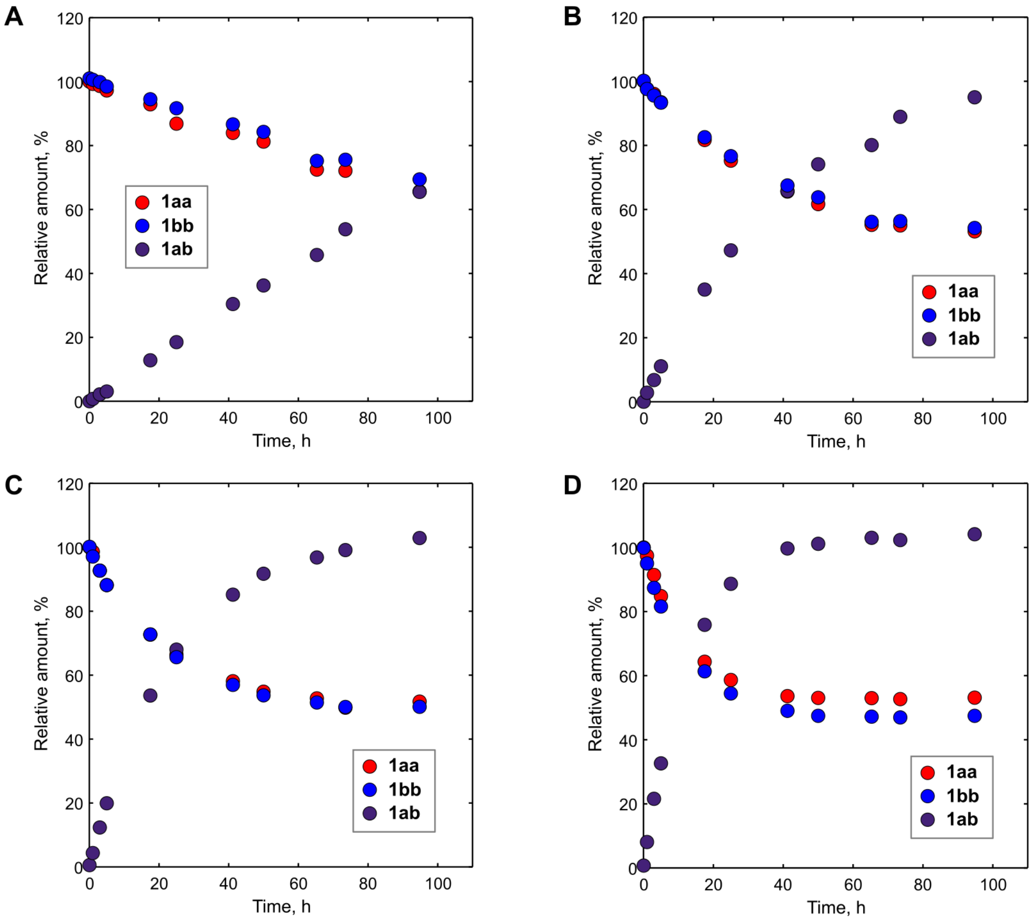

Figure S14: Kinetic plots of the amidinium metathesis between **1aa** and **1bb** in the presence of 100 mol% NBu_4OAc and varying amounts of **$BnNH_2$**: A) 0 mol%; B) 2.5 mol%; C) 5 mol%; D) 10 mol% (all relative quantities are with respect to the total amount of the amidinium species). Reaction conditions: 1.0 eq. **1aa** (10 μmol), 1.0 eq. **1bb** (10 μmol), 0–0.2 eq. **$BnNH_2$** (0–2 μmol), 2.0 eq. NBu_4OAc (20 μmol), solvent – MeCN (total volume – 400 μL), r.t. ***Comment***: the metathesis reaction containing 0.05% H_2O (50 mol% with respect to the total amount of the amidinium species) did not take place *in the absence* of both **$BnNH_2$** and NBu_4OAc. This disproves the hypothesis that the residual water from NBu_4OAc was the sole reason for the observed metathesis in case of (A). It is, however, possible that AcO^- catalyzes both the hydrolysis reaction and the amidinium exchange (see Fig. 3 in the main text). Therefore, we wondered if varying the water content in the reaction mixture while keeping the concentration of AcO^- fixed would affect the metathesis rate (Figure S15).

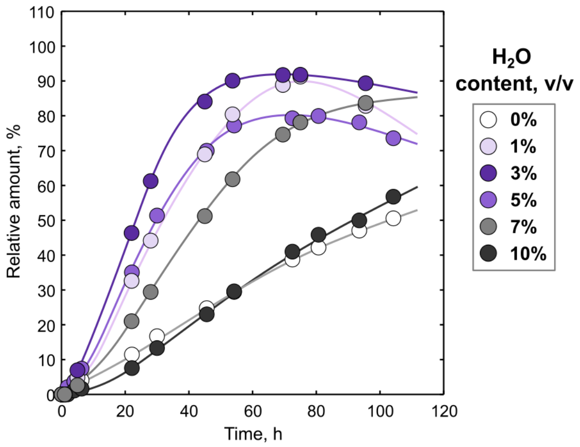

Figure S15: Kinetic plots of the amidinium metathesis between **1aa** and **1bb** in the presence of 100 mol% NBu_4OAc (with respect to the total amount of the amidinium species) and varying amounts of water. The lines are shown to guide the eye. Reaction conditions: 1.0 eq. **1aa** (10 µmol), 1.0 eq. **1bb** (10 µmol), 2.0 eq. NBu_4OAc (20 µmol), solvent – MeCN/H_2O (total volume – 400 µL), r.t. ***Comment***: Even 1% (v/v) of water significantly increased the rate of the metathesis. This result indicates that AcO^- might facilitate hydrolysis of the starting materials which, in turn, leads to release of free benzylamines that drive the metathesis. Therefore, even without addition of extra $BnNH_2$, the metathesis could take place in non-anhydrous MeCN in the presence of AcO^- (Fig. S14,A). Large amounts of water (>7% v/v) reduced the observed rate enhancement, possibly, for the same reasons as for the amidinium exchange in aqueous or alcoholic solvents (see Table S1 and discussion in the main text).

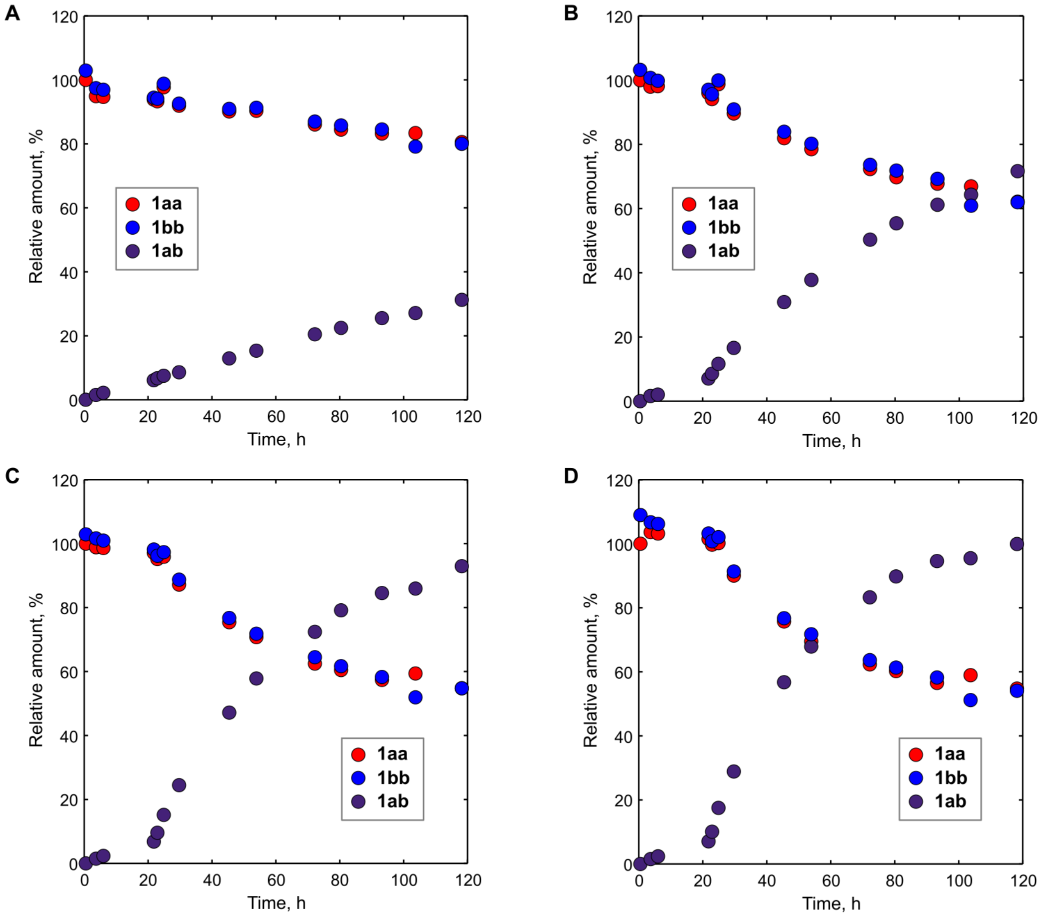

Figure S16: Kinetic plots of the amidinium metathesis between **1aa** and **1bb** in the presence of 4 mol% $BnNH_2$ and varying amounts of NBu_4OAc: A) 0 mol%; B) 20 mol%; C) 50 mol%; D) 90 mol% (all relative quantities are with respect to the total amount of the amidinium species). The acetate was added after 22 h from the reaction start. Reaction conditions: 1.0 eq. **1aa** (10 µmol), 1.0 eq. **1bb** (10 µmol), 0.075 eq. $BnNH_2$ (0.75 µmol), 0–1.8 eq. NBu_4OAc (0–18 µmol), solvent – MeCN (total volume – 400 µL), r.t.

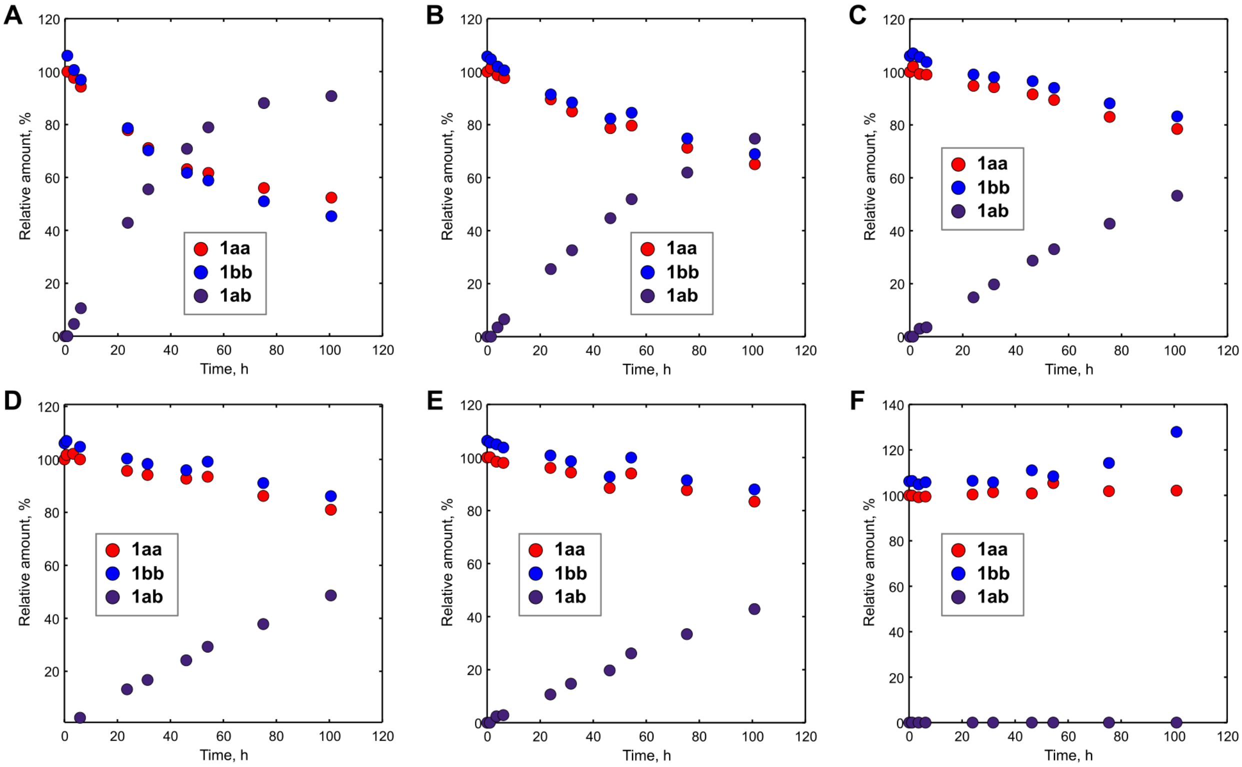

Figure S17: Kinetic plots of the amidinium metathesis between **1aa** and **1bb** in the presence of 5 mol% **BnNH$_2$** and different anions (as NBu_4^+ salts, 100 mol%): A) $H_2PO_4^-$; B) Cl^-; C) I^-; D) PF_6^-; E) NO_3^-; F) HSO_4^- (all relative quantities are with respect to the total amount of the amidinium species). Reaction conditions: 1.0 eq. **1aa** (10 μmol), 1.0 eq. **1bb** (10 μmol), 0.1 eq. **BnNH$_2$** (1 μmol), 2.0 eq. NBu_4^+ salt (20 μmol), solvent – MeCN (total volume – 400 μL), r.t. In case of metatheses in the presence of $H_2PO_4^-$ (A) and Cl^- (B), the solvent contained 20% and 10% (v/v) MeOH respectively (to increase solubility of the amidinium salts).

Table S3: Half-lives of the metathesis between **1aa** and **1bb** in the presence of different anions and substoichiometric amounts of **$BnNH_2$** (combined data from Figures S13, S14 and S17).

Entry	Anion	$BnNH_2$, mol%	$t_{1/2}$, min[a]
1	BPh_4^-	0	∞
2	AcO^-	0	70
3	BPh_4^-	2.5	>330[b]
4	AcO^-	2.5	27
5	BPh_4^-	5	102
6	AcO^-	5	16
7	BPh_4^-	10	32
8	AcO^-	10	9
9	$H_2PO_4^-$	5	28
10	Cl^-	5	54
11	I^-	5	92
12	PF_6^-	5	100
13	NO_3^-	5	114
14	HSO_4^-	5	∞

[a] The time when the amount of metathesis product **1ab** reaches 50% from its equilibrium amount.
[b] The value was obtained by linear extrapolation of the obtained experimental data (linear extrapolation underestimates the actual reaction half-life).

5 NMR spectra

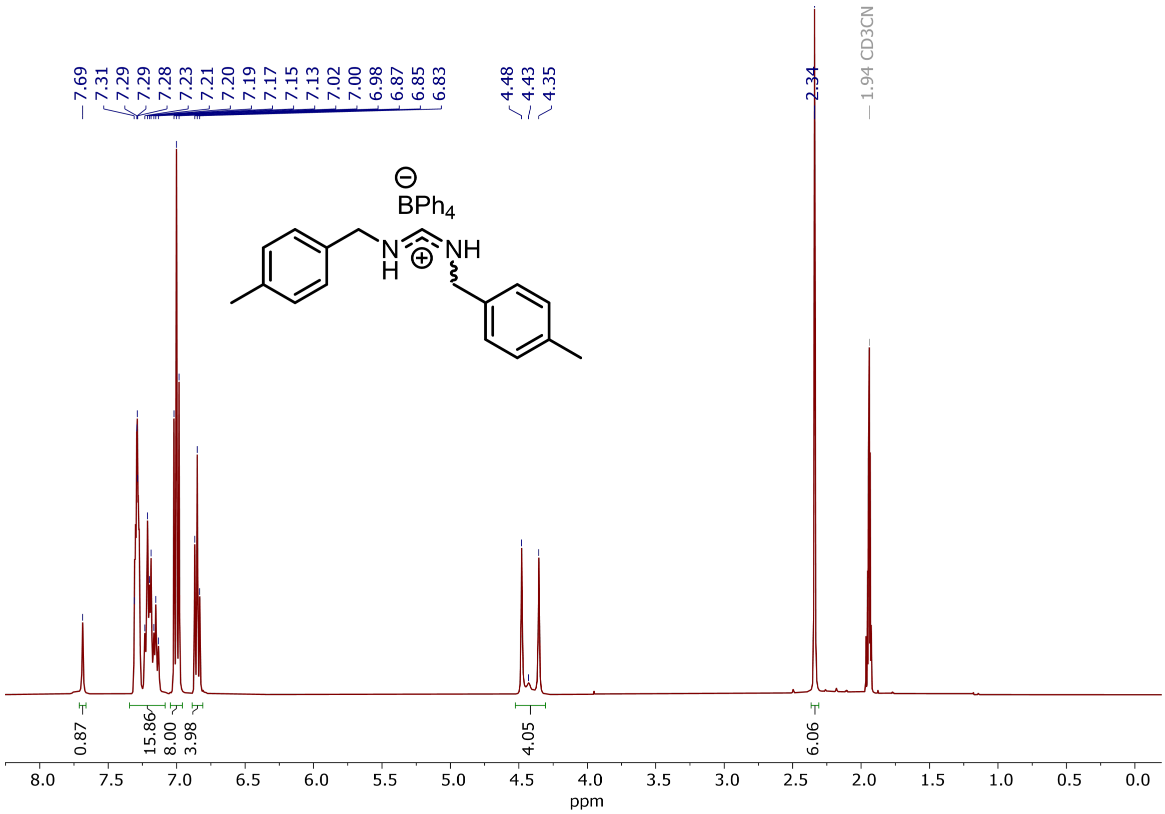

Figure S18: ^{1}H NMR spectrum (400 MHz, CD_3CN, 295 K) of **1bb**·BPh_4.

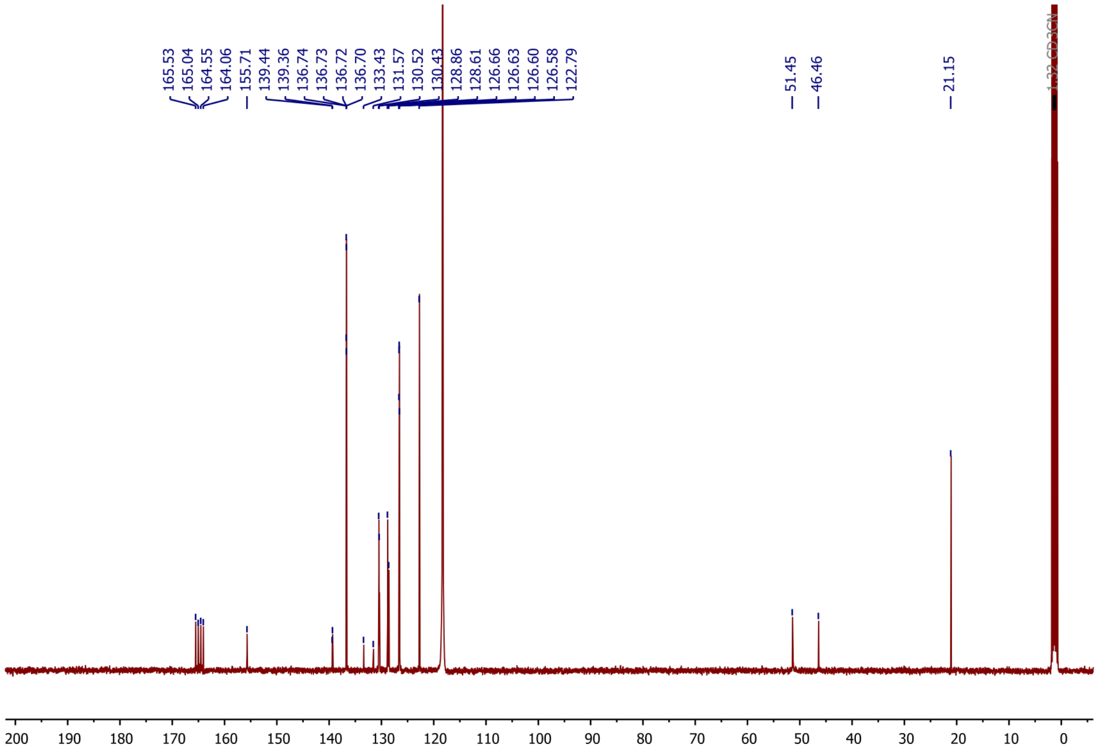

Figure S19: ^{13}C NMR spectrum (101 MHz, CD_3CN, 298 K) of **1bb**·BPh_4.

References

[1] O. Borodin, Y. Shchukin, C. C. Robertson, S. Richter, M. von Delius, *J. Am. Chem. Soc.* **2021**, *143*, PMID: 34559523, 16448–16457, DOI 10.1021/jacs.1c05230.
